# Perioperative chemotherapy with nivolumab for HER2-negative locally advanced gastric cancer: a case series

**DOI:** 10.1186/s40792-024-02001-w

**Published:** 2024-08-28

**Authors:** Yuta Toji, Shintaro Takeuchi, Yuma Ebihara, Yo Kurashima, Kazuaki Harada, Mariko Hayashi, Hirotake Abe, Hideyuki Wada, Satoko Yorinaga, Toshiaki Shichinohe, Utano Tomaru, Yoshito Komatsu, Satoshi Hirano

**Affiliations:** 1 Department of Gastroenterological Surgery II, Division of Surgery, Faculty of Medicine, Hokkaido University, West-7, North-15, Kita-ku, Sapporo, Hokkaido 060-8638 Japan; 2 Department of Gastroenterology and Hepatology, Faculty of Medicine, Hokkaido University, West-7, North-15, Kita-ku, Sapporo, Hokkaido 060-8648 Japan; 3 Department of Surgical Pathology, Hokkaido University Hospital, West-5, North-14, Kita-ku, Sapporo, Hokkaido 060-8648 Japan; 4 Department of Cancer Center, Hokkaido University Hospital, West-5, North-14, Kita-ku, Sapporo, Hokkaido 060-8648 Japan

**Keywords:** Gastric cancer, Nivolumab, Perioperative chemotherapy

## Abstract

**Background:**

Nivolumab with chemotherapy has been transformative for metastatic gastric cancer (GC). The potential of this regimen for local tumor control could be utilized for perioperative chemotherapy in locally advanced GC with bulky tumors or lymph node metastasis involving other organs.

**Case presentation:**

Five patients with HER2-negative advanced GC were treated with nivolumab and oxaliplatin-based chemotherapy. All patients presented with clinical stage III or IVA GC with tumors in contact with either the pancreas or liver. Following chemotherapy, all tumors demonstrated shrinkage, allowing successful radical gastrectomies including four minimally invasive approach without postoperative complications. Four patients avoided combined resection of other organs.

**Conclusions:**

Perioperative chemotherapy with nivolumab was effective for local disease control in this case series. This regimen could be a promising treatment approach for locally advanced GC; however, its survival benefits should be evaluated in clinical trials.

## Background

Surgical treatment of locally advanced gastric cancer (GC), especially for patients with bulky tumors extending into adjacent organs, remains debatable in clinical settings [1]. Current imaging studies cannot accurately diagnose whether a tumor invades adjacent organs at the histopathological level [2, 3]. Some studies have demonstrated the feasibility of upfront surgery with multivisceral resection (MVR) for selected cases [4–6]; however, this approach is associated with higher postoperative morbidity, especially in gastrectomies involving pancreatectomy [7–10]. In addition, some patients who undergo upfront surgery may lose the opportunity to receive adjuvant chemotherapy (AC) owing to lower chemotherapy tolerance caused by reduced oral intake and body weight loss [11–13].

Perioperative chemotherapy for resectable GC has been widely accepted in Western countries [14, 15]. Similarly, a recent clinical trial in Asian patients has reported well-tolerated results [16]; however, its inclusion criteria, such as disease stage and biomarkers, differed among institutions or countries [17]. Perioperative chemotherapy has several advantages, particularly for clinical T4 (cT4) or cN + disease, where tumor downstaging may contribute to a higher R0 resection rate and the avoidance of MVR. Thus, locally advanced GC in contact with other organs may be a potential target for perioperative chemotherapy.

Nivolumab combined with chemotherapy has shown practice-changing results for unresectable advanced or metastatic GC [18, 19]. Consequently, the introduction of immune-checkpoint inhibitors (ICIs) as a preoperative treatment for GC is currently underway [20–22]. Given the relatively high objective response rate of this regimen [18], it is also applicable as a preoperative treatment for resectable locally advanced GC.

Herein, we report five cases of human epidermal growth factor receptor 2 (HER2)-negative advanced GC treated with nivolumab and oxaliplatin-based chemotherapy. Our discussion focuses on the efficacy of this regimen in reducing surgical invasiveness through tumor downstaging.

## Case presentation

### A representative case

**Fig. 1:**
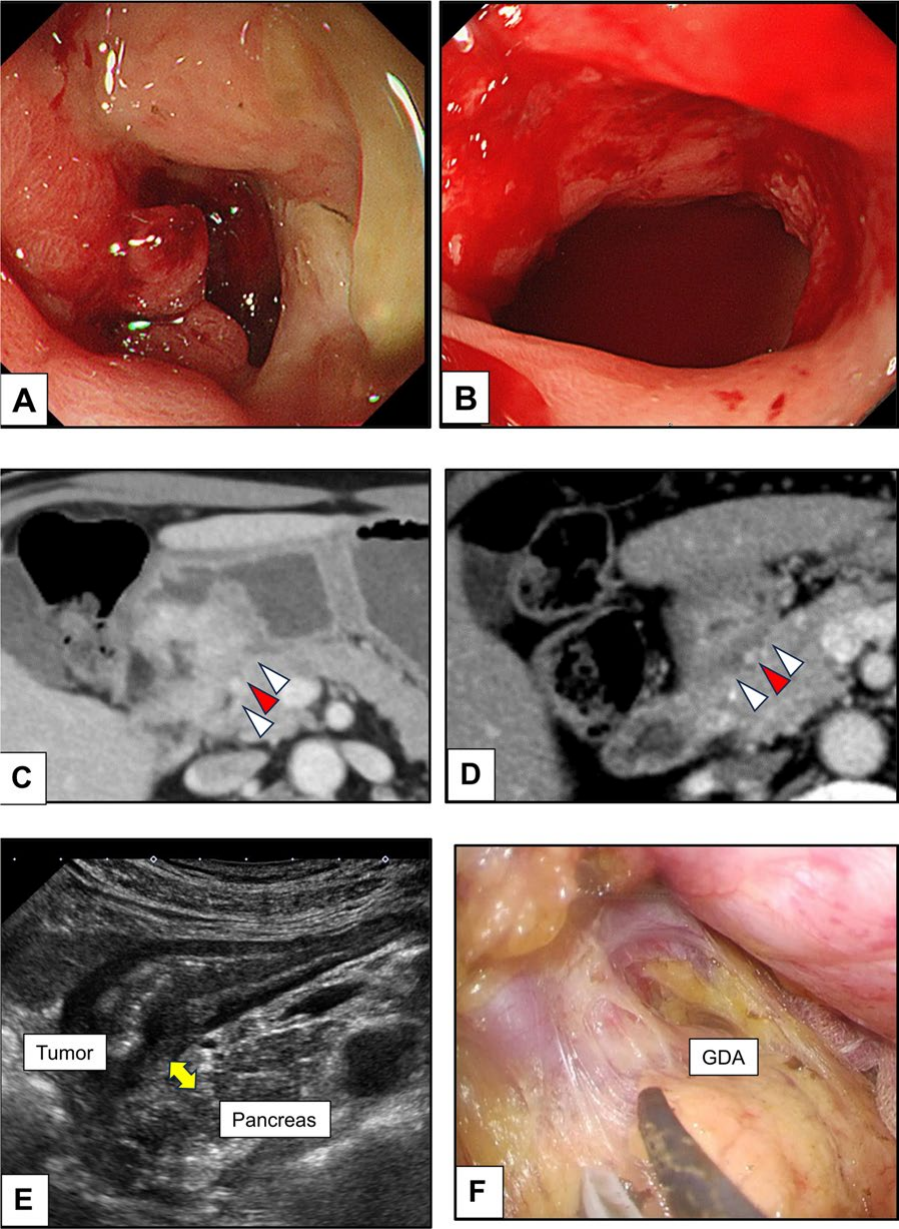
Findings of Case 1. **A** Gastric cancer extending from the pylorus to the duodenal bulb. **B** Tumor has shrunk after four courses of perioperative chemotherapy. **C** Tumor is in close contact with the pancreas (white arrowhead) and the gastroduodenal artery (GDA) (red arrowhead). **D** Area between the tumor and the pancreas contains a single layer of low-density area after perioperative chemotherapy. **E** Abdominal ultrasound indicating the presence of a space between the tumor and pancreas (yellow arrow). **F** Area between the tumor and the pancreas shows only edematous changes

**Fig. 2:**
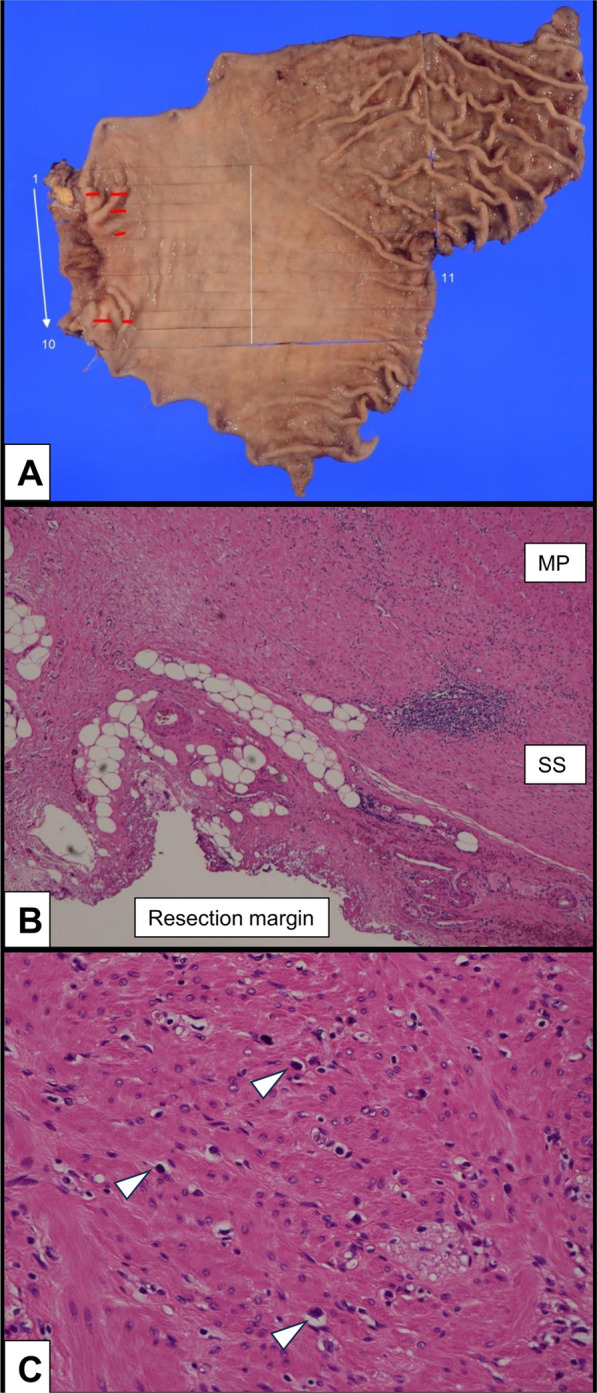
Pathological examinations of Case 1. **A** Macroscopic observation of the tumor (red line). **B** Microscopic examination reveals that the tumor is largely absent and the resection margin is negative. **C** Almost none of the viable tumor cells (white arrowhead) remain in the tumor area, and the histological evaluation is Grade 2

A 62-year-old man (Case 1 in Tables 1, 2, 3) presented with epigastric pain, and upper endoscopy revealed a tumor extending from the pylorus to the duodenal bulb (Fig. 1A). Signet-ring cells were detected by endoscopic tissue biopsy, and the programmed cell death ligand 1 (PD-L1) combined positive score (CPS) was less than 1. Initial computed tomography (CT) revealed the main tumor lesion in close contact with the pancreatic head and gastroduodenal artery (Fig. 1C). We diagnosed advanced GC classified as cT4b(panc)cN0cM0, clinical stage (cStage) IVA according to the Union for International Cancer Control TNM classification of malignant tumours, 8th edition, suggesting a possible need for pancreatoduodenectomy for R0 resection. Considering local factors, surgical invasiveness, and potentially poor outcomes, induction chemotherapy instead of upfront surgery was selected. After four cycles of the folinic acid, fluorouracil, and oxaliplatin (FOLFOX) with nivolumab, the primary tumor significantly regressed and it detached from the pancreas, indicating successful tumor downstaging (Fig. 1B–E). We performed a laparoscopic distal gastrectomy with D2 lymph node dissection. Intraoperatively, we observed only edematous changes in the posterior side of the tumor (close to the pancreatic head), and pancreatoduodenectomy was avoided (Fig. 1F). The postoperative course was uneventful, and the patient was discharged on postoperative day (POD) 8. The pathological diagnosis confirmed gastric cancer, classified as ypT3(SS)ypN0ycM0, ypStage IIA with distal and resection margins free of cancer cells (Fig. 2A), and Grade 2 histological treatment effect (Fig. 2B, C). AC with S-1 and oxaliplatin (SOX) regimen was initiated on POD 33; however, the treatment was discontinued after one cycle owing to an oxaliplatin-induced hypersensitivity reaction and the patient’s intolerance. At 6 month post-surgery, blood examination revealed asymptomatic hypothyroidism as an immune-related adverse event (irAE), necessitating initiation of thyroid hormone replacement therapy. The patient remained without recurrence on subsequent imaging follow-up at 14 month post-surgery.

**Table 1:** Patient characteristics

Case	Age	Sex	Histological classification^a^	CPS^b^	HER2	Pre-IC cTNM^c^	Pre-IC cStage^c^	Post-IC ycTNM^d^	Post-IC ycStage^d^
1	62	M	por2 > > sig	CPS < 1	Negative	T4bN0M0	IVA	T3N0M0	IIB
2	65	M	por2 > por1 + tub2	1 < CPS < 5	Negative	T4aN2M0	III	T4aN1M0	III
3	81	M	por1	CPS ≥ 5	Negative	T4aN2M0	III	T4aN1M0	III
4	60	M	por1 > > tub2	not examined	Negative	T4bN0M0	IVA	T4aN0M0	IIB
5	72	M	por	CPS ≥ 10	Negative	T4bN1M0	IVA	T3N0M0	IIB

*por1* poorly differentiated adenocarcinoma, solid type, *por2* poorly differentiated adenocarcinoma, non-solid type, *sig* signet-ring cell carcinoma, *tub2* tubular adenocarcinoma, moderately differentiated type, *CPS* combined positive score, *HER2* human epidermal growth factor receptor 2, *IC* induction chemotherapy

^a^Histological classification: Japanese classification of gastric carcinoma, 3rd English edition [23]

^b^CPS: score of programmed cell death ligand 1 expression in tumors, calculated by immunohistochemistry

^c^cTNM/cStage: clinical classification according to the Union for International Cancer Control TNM classification of malignant tumours, 8th edition

^d^ycTNM/ycStage: clinical classification after perioperative therapy according to the Union for International Cancer Control TNM classification of malignant tumours, 8th edition

**Table 2:** Summary of treatments

Case	Reason of IC	IC regimen	Total cycle of IC	Cycle of IC with Nivo	Procedure	Operation time (min)	Blood loss (ml)	AC regimen	Cycle of AC	irAE
1	Tumor contact to pancreas	FOLFOX + Nivo	4	3	LDG	465	150	SOX	1	HYPOTHYROIDISM
2	Lymph contact to pancreas	SOX + Nivo	3	2	LTG	424	75	SOX	5	no
3	Lymph contact to liver	SOX + Nivo	3	2	DG	289	245	non-AC	–	no
4	Tumor contact to liver	SOX + Nivo	5	2	LDG + Partial hepatectomy	362	40	SOX	2	Adrenal insufficiency
5	Tumor contact to pancreas	SOX + Nivo	4	2	RDG	273	5	not yet	–	no

*IC* induction chemotherapy, *FOLFOX* folinic acid, fluorouracil, and oxaliplatin, *SOX* S-1 and oxaliplatin, *Nivo*, nivolumab, *LDG* laparoscopic distal gastrectomy, *RDG* robotic distal gastrectomy, *LTG* laparoscopic total gastrectomy, AC adjuvant chemotherapy, *irAE* immune-related adverse events

**Table 3:** Pathology and short-term outcomes

Case	RECIST^a^	Residual tumor	Histological evaluation^b^	ypTNM^c^	ypStage^c^	Follow-up period (month)	Recurrence
1	PR	R0	Grade 2	T3N0M0	IIA	14	NO
2	PR	R0	Grade 2	T3N2M0	IIIA	11	NO
3	PR	R0	Grade 2	T2N1M0	IIA	4	NO
4	PR	R0	Grade 1b	T2N0M0	IB	2	NO
5	PR	R0	Grade 3	T0N0M0	0	1	NO

*RECIST*, Response Evaluation Criteria in Solid Tumors, *PR* partial response

^a^RECIST version 1.1 [24]

^b^Histological evaluation: histological evaluation criteria for tumor response after preoperative therapy based on the Japanese classification of gastric carcinoma-3rd English edition [23]

^c^ypTNM/ypStage: pathological classification after perioperative therapy according to the Union for International Cancer Control TNM classification of malignant tumours, 8th edition

### Summary of five cases

We treated five consecutive locally advanced GC cases using nivolumab combined with chemotherapy (Tables 1 and 2). The short-term outcomes are listed in Table 3.

**Fig. 3:**
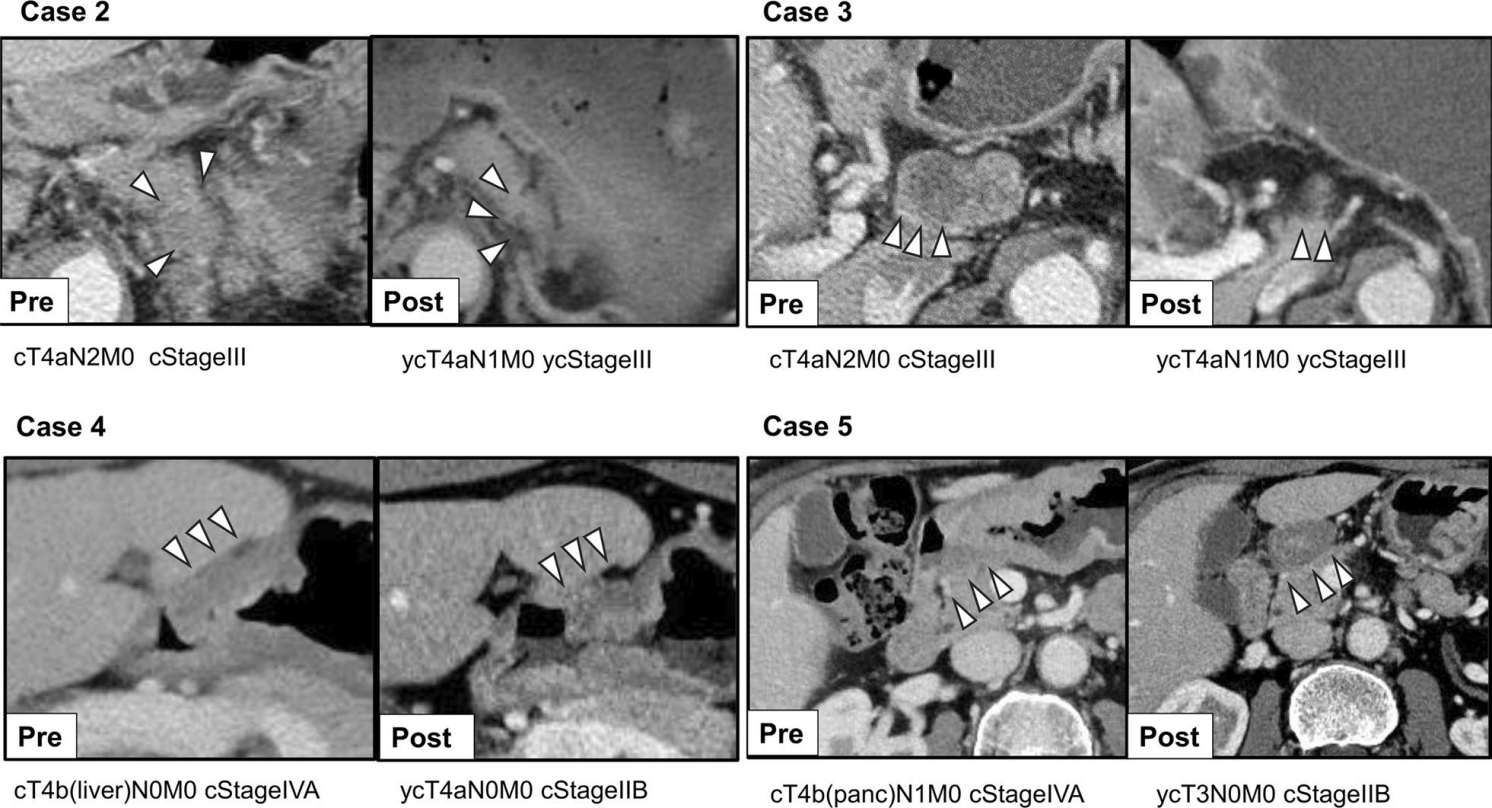
Comparison of computed tomography (CT) scans before and after perioperative chemotherapy. We administered perioperative chemotherapy with nivolumab for locally advanced gastric cancer. The indications for perioperative chemotherapy were as follows: lymph node adjacency to the pancreas (Case 2), lymph node adjacency to the liver (Case 3), tumor adjacency to the liver (Case 4), and tumor adjacency to the pancreas (Case 5). An objective response with major tumor shrinkage was observed in all patients

All patients had clinical stage III or IVA GC (poorly differentiated carcinoma) with bulky primary lesions or lymph node metastasis in contact with adjacent organs (three and two cases involving the pancreas and liver, respectively) without distant metastasis (Fig. 3). All cases were resectable GC; however, we selected induction chemotherapy as the treatment protocol, considering the patients’ local and oncological features, instead of upfront surgery with MVR. PD-L1 expression was assessed for four cases using CPS; however, only two cases showed moderate to strong expression (Cases 3 and 4; CPS ≥ 5). Immunohistochemical assessment of HER2 expression was negative in all the cases. Microsatellite instability (MSI) was evaluated only for Case 5, which was microsatellite stable. Preoperative chemotherapy included an oxaliplatin-based doublet regimen (FOLFOX or SOX) combined with nivolumab. The timing of gastrectomy was decided based on the follow-up CT findings; consequently, the duration of chemotherapy was 2–3 months, and the interval between chemotherapy and surgery was approximately 1 month. Staging laparoscopy was not performed because of a restricted schedule. The patient characteristics and treatment details, including the chemotherapy regimen and surgical procedure, are summarized in Tables 1 and 2.

**Fig. 4:**
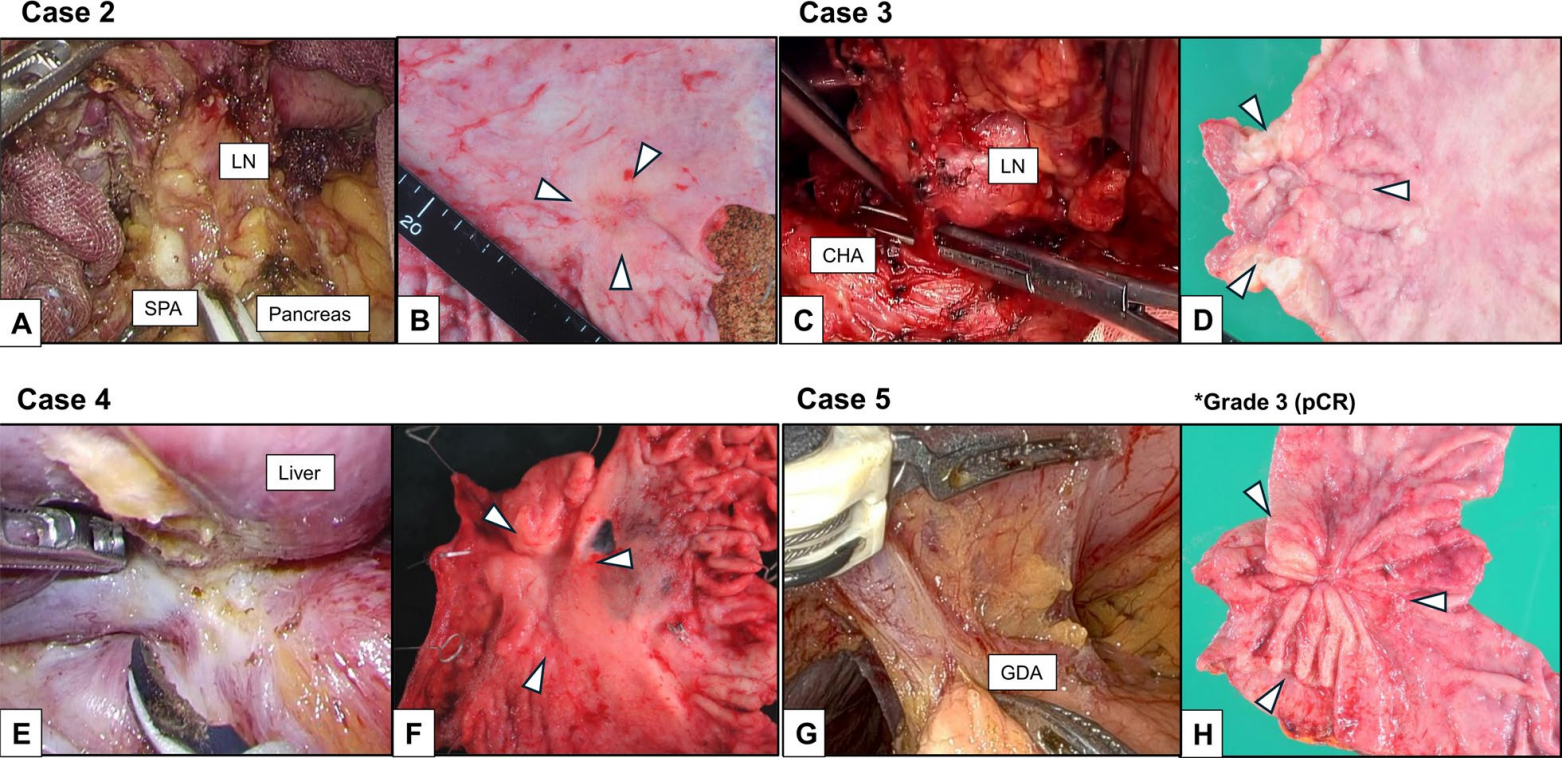
Operative findings and macroscopic observation. The lymph node metastases are adjacent to the pancreas and splenic artery in Case 2, or to the caudate lobe and common hepatic artery in Case 3; however, these were successfully dissected. The area between the tumor and pancreas shows only edematous changes in Case 5. The tumor invaded the liver, and it was necessary to perform a partial liver resection in Case 4. Macroscopic observation reveals tumor shrinkage in all cases. *Case 5 achieved Grade 3 effect (pCR: pathologically complete response)

Notably, the size of the bulky tumor lesions commonly decreased, and the contact between the tumor edges and adjacent organs became indistinct (Fig. 3). All patients showed partial response according to the Response Evaluation Criteria in Solid Tumors on posttreatment CT. The surgical teams carefully discussed the operative procedures, assessing the need for pancreatectomy or liver resection intraoperatively. The pancreas and liver were preserved in four cases owing to the absence of significant invasion noted during surgery. Partial liver resection was performed in one patient where GC had invaded the liver (Case 4). No postoperative complications occurred in any of the patients. R0 resection was achieved in all cases and tumor responses were observed by histological evaluation, including complete response in one patient (Case 5). After surgery, three patients received AC with the SOX regimen, one patient planned to receive AC (Case 5), and one patient declined AC upon request (Case 3). None of the patients experienced recurrence. The operative findings and macroscopic observation of Case 2–5 are summarized in detail (Fig. 4).

Two patients experienced irAEs, which were neither severe nor lethal, classified as Grade 2 according to the Common Terminology Criteria for Adverse Events. These events included hypothyroidism following AC in Case 1 and adrenal insufficiency during chemotherapy in Case 2, necessitating hormone replacement therapy. Short-term outcomes including the safety and efficacy of this regimen are listed in Table 3.

## Discussion

In this case series, we report our experiences regarding five patients with HER2-negative advanced GC treated with nivolumab combined with chemotherapy. All patients had potentially resectable GC diagnosed as cT4b or cN2. A common feature among these cases was imaging evidence of gastric tumor or lymph node metastasis in close contact with the adjacent organs (three and two cases involving invasion by tumor and lymph node metastasis, respectively), necessitating extended surgery with MVR to achieve R0 resection [2, 25]. All tumors shrank effectively after two or three cycles of induction chemotherapy, and R0 resection was possible without MVR. This case series suggest the high local control rates of this regimen are beneficial in the role of perioperative chemotherapy for advanced GC.

In Japan, upfront gastrectomy and D2 lymph node dissection followed by AC are standard treatments for advanced GC [26]. However, the prognosis of highly advanced diseases, such as cT4 and cN2, remains dismal [27]. In the American Joint Committee on Cancer staging system, cT4b disease is classified as stage IV owing to its median survival of 8.4 months [28]. While some studies have showed the effectiveness of MVR for T4b GC including pancreatoduodenectomy for margin-free resection [4–6], the increased risk of postoperative complication and mortality cannot be ignored. Some studies indicate that pancreatic resection for GC may not improve oncological outcome [9, 10]. Thus, multimodal treatment is essential for improving the prognosis of locally advanced GC involving adjacent organs [13, 29]. We regarded these types of GCs as “borderline resectable” or “unresectable,” which required intensive preoperative chemotherapy in order to undergo radial surgery [30]. Consequently, the five cases presented herein were treated by a certain kind of “conversion surgery” strategy [31].

The specific inclusion criteria and perioperative chemotherapy regimens for GC have been evaluated in several studies [17]. Currently, cT3/4 and cN + are suggested as the optimal inclusion criteria for neoadjuvant chemotherapy (NAC) to detect pStage III GC in Japan [32]. A South Korean study has shown that cT4Nany may help select optimal patients for NAC [33]. The five cases in the present report met these criteria, indicating that these patients can benefit from intensive preoperative chemotherapy. In particular, we addressed the tumor-shrinkage effect of chemotherapy to reduce surgical invasiveness. Effective chemotherapy created a potential margin-free distance from other organs (Fig. 1E, F) and enabled organ preservation.

Based on the results of international clinical trials, nivolumab combined with chemotherapy has become a first-line chemotherapeutic treatment for unresectable advanced or metastatic GC [19, 20]. These studies suggest that 2–3 months of induction chemotherapy is reasonable, considering that tumors shrink within this timeframe with nivolumab combined with chemotherapy for unresectable advanced or metastatic GC [34]. In addition, conversion surgery for metastatic GC after a major response to nivolumab combined with chemotherapy has also been reported [35–37]. The findings of these reports and our five cases indicate that this regimen can be effective as induction therapy owing to its powerful downstaging potential, as reported for other cancers [38–40]. In the new era, biomarkers such as CPS, deficient mismatch repair/MSI-high (dMMR/MSI-H), and tumor mutation burden will be essential for selecting effective perioperative chemotherapy regimens, whether chemotherapy or ICIs [41]. In this case series, nivolumab combined with chemotherapy had a universal effect, regardless of the CPS score (Table 2). Similarly, the objective response rate was reportedly higher, regardless of the CPS, in patients who received nivolumab combined with chemotherapy than in those who received chemotherapy alone in the Checkmate 649 trial [42]. Therefore, nivolumab combined with chemotherapy can be utilized, regardless of the CPS, in patients with locally advanced GC to achieve tumor shrinkage and avoid MVR.

Selecting an appropriate chemotherapy regimen for use in combination with nivolumab is controversial. Fluorouracil, leucovorin, oxaliplatin, and docetaxel (FLOT) is a major perioperative chemotherapy regimen for GC worldwide, and evaluating its use in combination with ICIs is under progress [15, 22]. A Chinese study comparing neoadjuvant FLOT and SOX for locally advanced GC found no significant differences between the two with respect to tumor regression or adverse effects [43]. We selected a nivolumab plus oxaliplatin-based doublet regimen including SOX as induction chemotherapy and three patients were treated with SOX as AC [44]. Currently, several perioperative clinical trials using various combinations of ICIs and chemotherapy for gastroesophageal cancer are ongoing with different designs and endpoints [21, 22, 45–47]. Although the latest KEYNOTE-585 data do not show survival benefits of using neoadjuvant ICIs for advanced GC, the pathological complete response rate was significantly higher in the ICI plus chemotherapy arm compared with the chemotherapy alone arm [21]. Thus, perioperative ICI use and chemotherapy for GC need to be tailored according to patient characteristics, biomarkers, as well as the targeted endpoints in the near future.

IrAEs are a concern with ICIs use [48]. Some studies on neoadjuvant immunotherapy in lung cancer have shown a low incidence of irAEs, mainly grade 1 or 2, with lower incidence of surgery delays or cancellations [38, 39]. Although two patients in this case series experienced grade 2 irAEs, the impact on treatment was minimal. Given the reports of severe irAEs [49–51], it is crucial to accumulate more evidence on the frequency and timing of irAEs when using nivolumab as perioperative chemotherapy for resectable GC. These issues need to be resolved before this regimen can be fully integrated as perioperative chemotherapy for GC.

Postoperative complications have been shown to negatively impact the survival outcomes of GC [11, 52–54]. Therefore, it is reasonable to reduce surgical invasiveness through preoperative downstaging using an effective perioperative chemotherapy regimen. This case series provides clinical insights into the potential of nivolumab combined with chemotherapy regimen as a choice of perioperative chemotherapy for GC, supported by detailed patient data and imaging findings.

The limitations of this report are that it was a small case series, and the follow-up duration was short. The efficacy of this perioperative chemotherapy regimen for GC should be evaluated in rigorous clinical trials including survival outcomes. Furthermore, whether all patients with advanced GC would benefit from this regimen is uncertain, as per the latest clinical trial data on neoadjuvant ICIs [21]. The routine use of nivolumab with chemotherapy for resectable GC is inappropriate, and it should be reserved for patients with poor outcomes despite standard treatment. We discussed the treatment plans of all patients with a multidisciplinary team, and informed consent for perioperative chemotherapy with nivolumab was obtained from all patients. This case series suggests that the treatment protocol used in our study is promising and suitable for the treatment of locally advanced GC in contact with other organs, at least for ensuring local disease control.

## Conclusions

Based on our five cases, perioperative chemotherapy combined with nivolumab for HER2-negative locally advanced GC in contact with adjacent organs shows potential, especially for local disease control. Clinical trials and further data accumulation are critically needed to investigate the survival outcomes.

## Data Availability

Not applicable.

## Abbreviations

GC: Gastric cancer
MVR: Multi-visceral resection
AC: Adjuvant chemotherapy
ICIs: Immune-checkpoint inhibitor
HER2: Human epidermal growth factor receptor 2
PD-L1: Programmed cell death ligand 1
CPS: PD-L1 combined positive score
CT: Computed tomography
FOLFOX: Folinic acid, fluorouracil and oxaliplatin
POD: Postoperative day
SOX: S-1 and oxaliplatin
irAE: Immune related adverse events
MSI: Microsatellite instability
NAC: Neoadjuvant chemotherapy
dMMR: Deficient mismatch repair
FLOT: Fluorouracil, leucovorin, oxaliplatin, and docetaxel
